# Obesity-related drug-metabolizing enzyme expression alterations in the human liver

**DOI:** 10.1016/j.biopha.2025.118155

**Published:** 2025-05-12

**Authors:** Katarzyna Kosicka-Noworzyń, Aleksandra Romaniuk-Drapała, Yi-Hua Sheng, Christine Yohn, Luigi Brunetti, Leonid Kagan

**Affiliations:** a Department of Physical Pharmacy and Pharmacokinetics, Poznan University of Medical Sciences, Rokietnicka 3, Poznań 60-806, Poland; b Department of Clinical Chemistry and Molecular Diagnostics, Poznan University of Medical Sciences, Rokietnicka 3, Poznań 60-806, Poland; c Department of Pharmaceutics, Ernest Mario School of Pharmacy, Rutgers, The State University of New Jersey, 160 Frelinghuysen Road, Piscataway, NJ 08854, USA; d Department of Pharmacy Practice and Administration, Ernest Mario School of Pharmacy, Rutgers, The State University of New Jersey, 160 Frelinghuysen Road, Piscataway, NJ 08854, USA; e Center of Excellence for Pharmaceutical Translational Research and Education, Ernest Mario School of Pharmacy, Rutgers, The State University of New Jersey, 160 Frelinghuysen Road, Piscataway, NJ 08854, USA

**Keywords:** Drug-metabolizing enzymes, Pharmacokinetics, Obesity, Hepatic clearance

## Abstract

**Objective:**

Implications of obesity extend beyond the association with various health conditions, impacting physiological changes that affect the liver and the activity of metabolizing enzymes. Given the prevalence of obesity and the risk for drug-drug interactions owing to the comorbidity burden, the current drug dosage recommendations may need reevaluation for patients with obesity. This study evaluated the implications of obesity on the gene expression of hepatic drug-metabolizing enzymes. As drug clearance is an essential pharmacokinetic parameter for maintaining drug dosing regimens, investigating alterations in metabolizing enzymes expression is a critical step.

**Methods:**

Human liver samples were collected post-mortem from 32 individuals and classified into the control (18.5 ≤ BMI <25 kg/m^2^; range 18.9–24.4 kg/m^2^; median 2^2^.3 kg/m^2^) and the study group (BMI ≥25 kg/m^2^; range 25.1–55.5 kg/m^2^; median 31.2 kg/m^2^). Real-time quantitative PCR was performed for the analysis of 168 drug-metabolizing enzymes.

**Results:**

Our studies revealed several potential physiologically relevant differences, but the statistical significance was reached only for *ALDH3B1*, *PTGS1*, and *CEL* (all being up-regulated in the study group).

**Conclusions:**

The study adds to our understanding of the mechanisms of pharmacokinetic changes in overweight and obesity. The findings require further exploration on the protein level, through proteomic and functional studies.

## 1. Introduction

The rising prevalence of overweight and obesity has become a global health concern. According to recent statistics, approximately 42 % of the US population suffer from obesity [1]. The implications of obesity extend beyond its association with various health conditions, impacting physiological changes with a significant effect on the liver [2]. The liver is pivotal in several key metabolic processes and plays a central role in transforming endogenous substrates, including drug biotransformation [3]. The main components involved in these processes are intracellular drug-metabolizing enzymes.

The liver undergoes intricate alterations in structure and function in response to obesity. One of the most common changes is an enhancement in cardiac output, which increases liver blood flow [4]. However, the increased risk of non-alcoholic fatty liver disease (NAFLD) results in steatosis or non-alcoholic steatohepatitis (NASH). In these conditions, fat-laden hepatocytes are swollen, which can lead to narrowing and distortion of the sinusoidal lumen, further restricting microvascular blood flow [5,6]. Obesity is associated with pathophysiological changes that may influence drug pharmacokinetics (PK). Hepatic drug metabolism depends on intrinsic liver clearance (Cl_int_), which is determined by enzyme activity and transporters in the liver [4]. Abnormal fat deposition and inflammation can influence hepatic metabolizing enzymes and drug transporters expression and/or activity [7–9].

Hepatic drug-metabolizing enzymes are responsible for the modification and conjugation of drugs [10]. Phase 1 metabolism, primarily mediated by cytochrome P450 enzymes (CYP), plays a pivotal role in drug oxidation. The CYP P450 superfamily encompasses 17 families and 39 subfamilies of enzymes, and many CYPs show the highest abundance in the human liver. The most important hepatic monooxygenases for drug pharmacokinetics in humans are CYP1A2, CYP2A6, CYP2B6, CYP2C8, CYP2C9, CYP2C19, CYP2D6, CYP2E1, CYP3A4, and CYP3A5 [11,12]. In phase 2 of drug metabolism, conjugation reactions are crucial in generating more soluble products that can be easily excreted into bile or urine. Uridine diphosphate glycosyltransferases (UGTs) play a major role in phase 2 biotransformation, taking part in glucuronidation. Notably, approximately 45 % of all metabolized drugs involve CYP3A4-mediated biotransformation, predominantly in hepatocytes and the gut wall. Studies have demonstrated that obesity influences drug metabolism pathways differentially [13]. While clearance of CYP3A4 substrates is lower in patients with obesity, drugs primarily metabolized by UGT, xanthine oxidase, N-acetyltransferase, or CYP2E1 exhibit higher clearance in individuals with obesity [14–17]. Importantly, the plasma clearance of a drug will depend on many factors, including expression and activity of the enzymes and transporters, size of a metabolizing organ, blood flow, etc.; each of which may change in obesity in their own way [18,19].

There has been a substantial increase in understanding of changes in PK and pharmacodynamics (PD) associated with obesity. Nonetheless, future research should address existing knowledge gaps, such as the need to reevaluate current drug dosage recommendations for patients with obesity, in order to optimize therapeutic interventions to enhance drug safety and efficacy. Due to the higher frequency of obesity-related comorbidities such as diabetes, hypertension, and dyslipidemias, patients are more often subjected to polypharmacy [20]. Our study aimed to screen for differences in the mRNA expression of 168 genes encoding metabolizing enzymes (phases 1 and 2) between patients with overweight/obesity and patients with a BMI within the range 18.5 – 24.9 kg/m2. This manuscript delves into the implications of obesity on the gene expression of hepatic drug-metabolizing enzymes. Given the prevalence of obesity and the significant risk for drug-drug interactions owing to the comorbidity burden, they evaluated alterations in drug-metabolizing gene expression as a first step is critical.

## 2. Methods

### 2.1. Human tissue specimens

Snap-frozen whole-liver human specimens from 32 deceased donors were obtained from the National Disease Research Interchange (NDRI) (Philadelphia, PA, USA). The Rutgers Biomedical Health Sciences Institutional Review Board approved this study as exempt research (Pro2019001020). Any tissue with signs of disease (i.e., malignancy, cirrhosis) was rejected.

As reported by the NDRI, the major cause of death of the patients included in the study was cardiac arrest or a related cardiac or cardiovascular event (cardiac arrest was reported for 15 patients, other cardiovascular reasons for 7 patients). Other reported causes of death were: respiratory failure – 5, trauma – 1, gunshot – 1, abdominal aorta aneurysm – 1, hemorrhage – 1, unspecified – 1. The clinical characteristics of the studied population are summarized in Table 1.

The donors of tissue specimens were divided into control and study groups based on their weight category. The control group included subjects of a healthy weight with a BMI of 18.5 – 25 kg/m^2^; the study group consisted of individuals with overweight and obesity (BMI ≥25 kg/m^2^). Before the assays, a single tissue block (from a single patient) was removed from −80 °C and placed in a weigh boat in dry ice. The liver specimens were on dry ice during this procedure and never allowed to thaw entirely.

### 2.2. RNA extraction and cDNA synthesis

RNA extraction was performed on approximately 30–35 mg of liver tissue using the AllPrep DNA/RNA/Protein Mini Kit (Qiagen, San Diego, CA, USA) and purified using the RNeasy Mini Kit (Qiagen, San Diego, CA, USA). Procedures were performed according to the manufacturer’s protocols. RNA quantity and quality were assessed using a NanoDrop 2000 (Thermo Scientific, Waltham, MA, USA). The yield was determined in duplicate at an absorbance of 260 nm. All samples showed 260/280 ratios exceeding 1.8, excluding protein presence. The cDNA (0.5 μg) was synthesized (in a reverse transcribed polymerase chain reaction (RT-PCR)) using a commercial RT2 First Strand Kit (Qiagen, San Diego, CA, USA) according to the manufacturer’s recommendations. The cDNA concentration was diluted, and 10 ng total RNA input was used per individual qPCR reaction.

### 2.3. Quantitative PCR

Gene-expression analysis of liver samples was conducted using the 96-well plates RT2 Profiler PCR Array Systems: Human Drug Metabolism: Phase 1 Enzymes, and Human Drug Metabolism: Phase 2 Enzymes (Qiagen, San Diego, CA, USA; product no. PAHS-068Z and PAHS-069Z). The complete gene list is available on Qiagen’s website (https://geneglobe.qiagen.com/us/product-groups/rt2-profiler-pcr-arrays) and summarized in Table S1 (supplementary file). qPCR was performed for the analysis of 168 genes of interest and 5 housekeeping genes, using a SYBR-Green-based method in 96-well plates (Qiagen, San Diego, CA, USA) in a QuantStudio 7 Pro Real-Time PCR instrument (Thermo Scientific, Waltham, MA, USA). Reference genes were selected using an online analysis tool, RefFinder [21], selection criteria for the most stable genes in obesity were described in detail [22]. Relative gene expression was normalized to the geometric means of housekeeping genes *RPLP0* and *GAPDH* and analyzed by the comparative threshold cycle method (C_t_). The cut-off C_t_ was set at 35 cycles for all analyses. Results are reported as the relative fold-change in gene expression between the control and study group, calculations were made using the ΔC_t_ method [23]. The fold-change was calculated by dividing the mean [2^∧^(−ΔC_t_)] values of the study group and the control group (specific values are available in Table S2 in the supplementary materials). Results were considered of potential biological relevance when the fold-change was ≥ 1.5 or ≤ −1.5.

### 2.4. Statistical analysis

There were 32 genes encoding metabolizing enzymes (14 genes of phase 1 enzymes and 18 genes of phase 2 enzymes; Fig. 2) for which the fold-change in mRNA expression was of potential biological relevance. For these genes, the distribution of log-transformed [2^∧^(−ΔC_t_)] values was tested for normality (Shapiro-Wilk test). Then, data without normal distribution were compared using the Mann-Whitney *U* test; normally distributed data were compared with the T-test (equal variances; F-test was performed to check for equality of variances) or the Welch test (unequal variances). No mathematical correction was used for multiple comparisons; a p-value< 0.05 was considered statistically significant. The Spearman’s rank test was applied to analyze the correlations between log-normalized [2^∧^(−ΔC_t_)] values and BMI (rho is the Spearman’s coefficient of rank correlation), and correlation was accepted at the significance level p = 0.05. Statistical analysis was performed using GraphPad Prism (GraphPad Prism 10, GraphPad Software, Inc., Boston, MA, USA) and MedCalc (version 22.030, MedCalc Software Ltd, Osten, Belgium).

## 3. Results

**Fig. 1.**
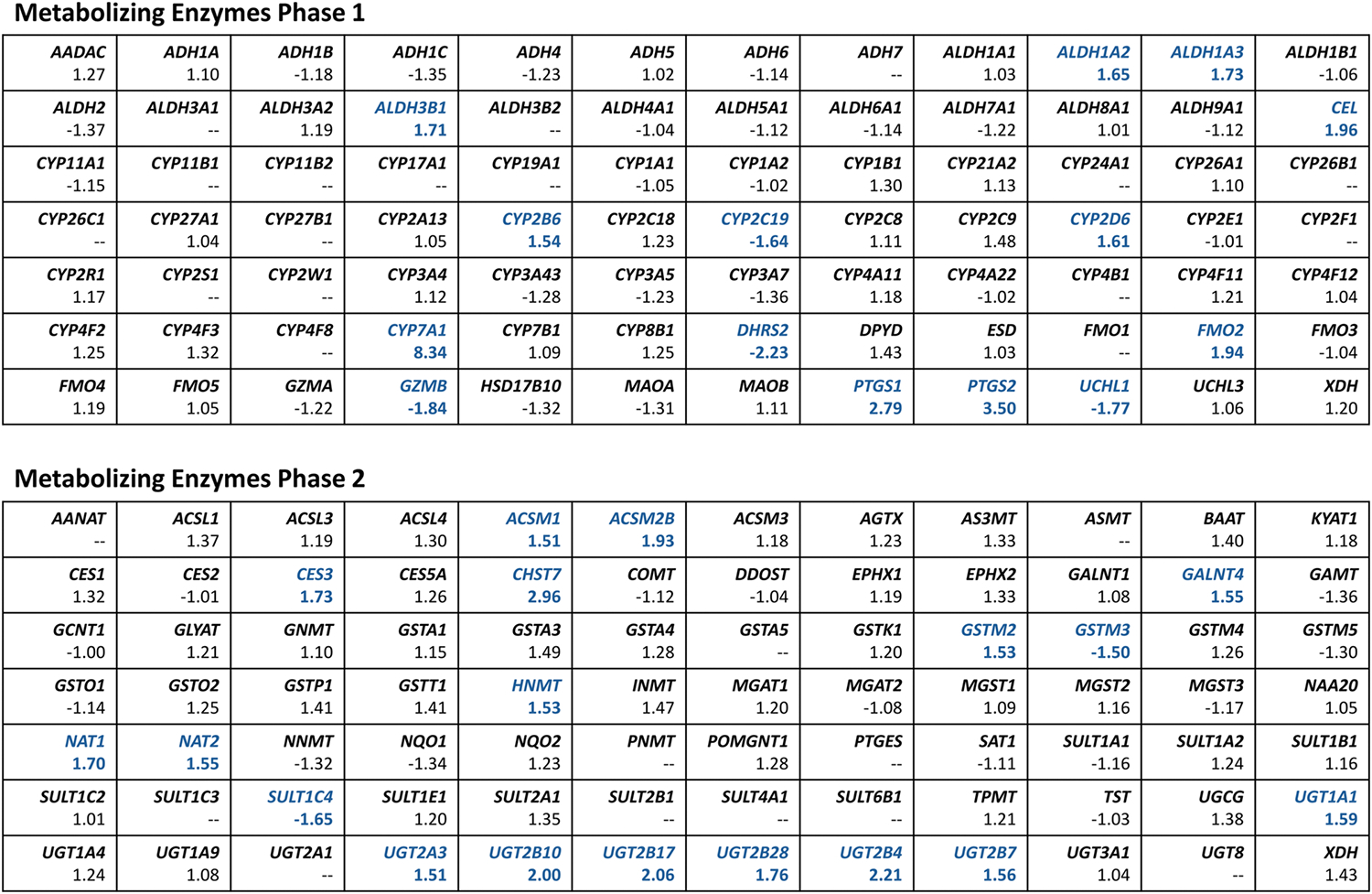
Drug-metabolizing enzyme genes of which the mRNA expression in human liver was evaluated, and the fold-change values. Each cell consists the gene abbreviation in bold (upper line); below the fold-change value is given for the liver tissue (lower line). Fold-change in gene expression between groups was calculated by dividing the respective mean [2^∧^(−ΔC_t_)] values (value for the study group divided by the value for the control group). Results were considered of potential biological relevance when the fold-change was ≥ 1.5 or ≤ −1.5; fold-change values above cut-off are marked blue.

**Fig. 2.**
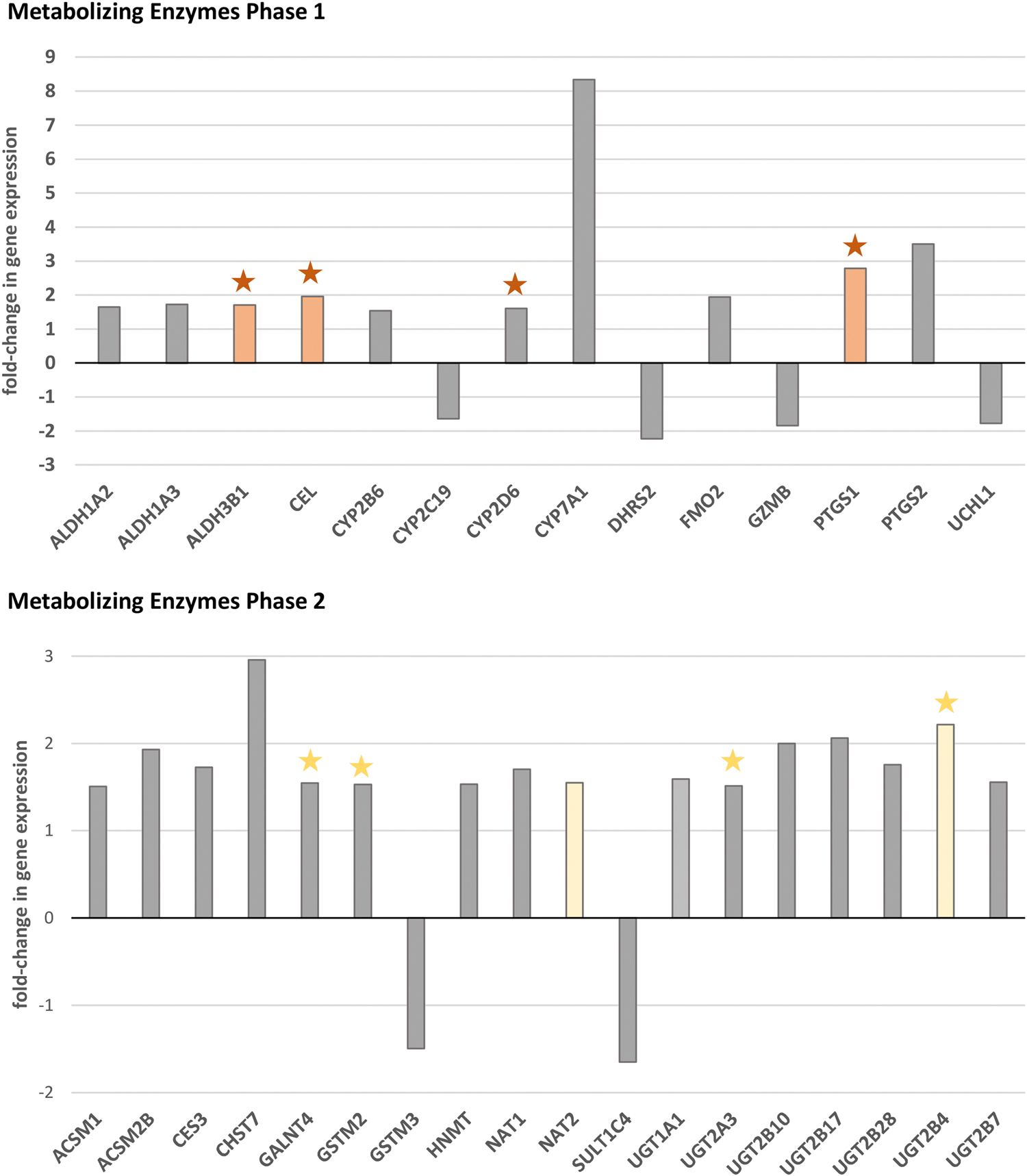
Differences in phase 1 and 2 metabolizing enzymes gene expression in the liver between overweight/obese (BMI ≥25) and lean (18.5 ≤ BMI <25) populations. Fold-change in gene expression between groups was calculated by dividing the respective mean [2^∧^(−ΔC_t_)] values. Results were considered of potential biological relevance when the fold-change was ≥ 1.5 or ≤ −1.5. Genes with a significant change (T-test or Mann-Whitney U-test; p < 0.05) in expression between groups are marked orange. Yellow color labels genes with a trend towards different expression (T-test or Mann-Whitney U-test; 0.05 ≤ p < 0.10). Grey color labels delineate results with no statistical significance (p > 0.10). Star indicates a correlation between the log-transformed [2^∧^(−ΔC_t_)] and BMI: orange star labels significant results (Spearman rank test, p < 0.05), yellow star indicates correlations close to significant (Spearman rank test, 0.05 <p < 0.10).

We evaluated the mRNA expression of 168 genes encoding phase 1 and phase 2 drug-metabolizing enzymes in the human liver (Table S1). Genes with expression below the cut-off (C_t_ >35) in more than 50 % of control or/and study group subjects were excluded from further analysis. Fold-change in hepatic gene expression was calculated for 140 genes (Fig. 1); the cut-off for biological relevance was set at 1.5 [24]. Fold-change values above the cut-off were noted for 14 genes of phase 1 metabolizing enzymes and 18 genes of phase 2 metabolizing enzymes (Fig. 2).

**Fig. 3.**
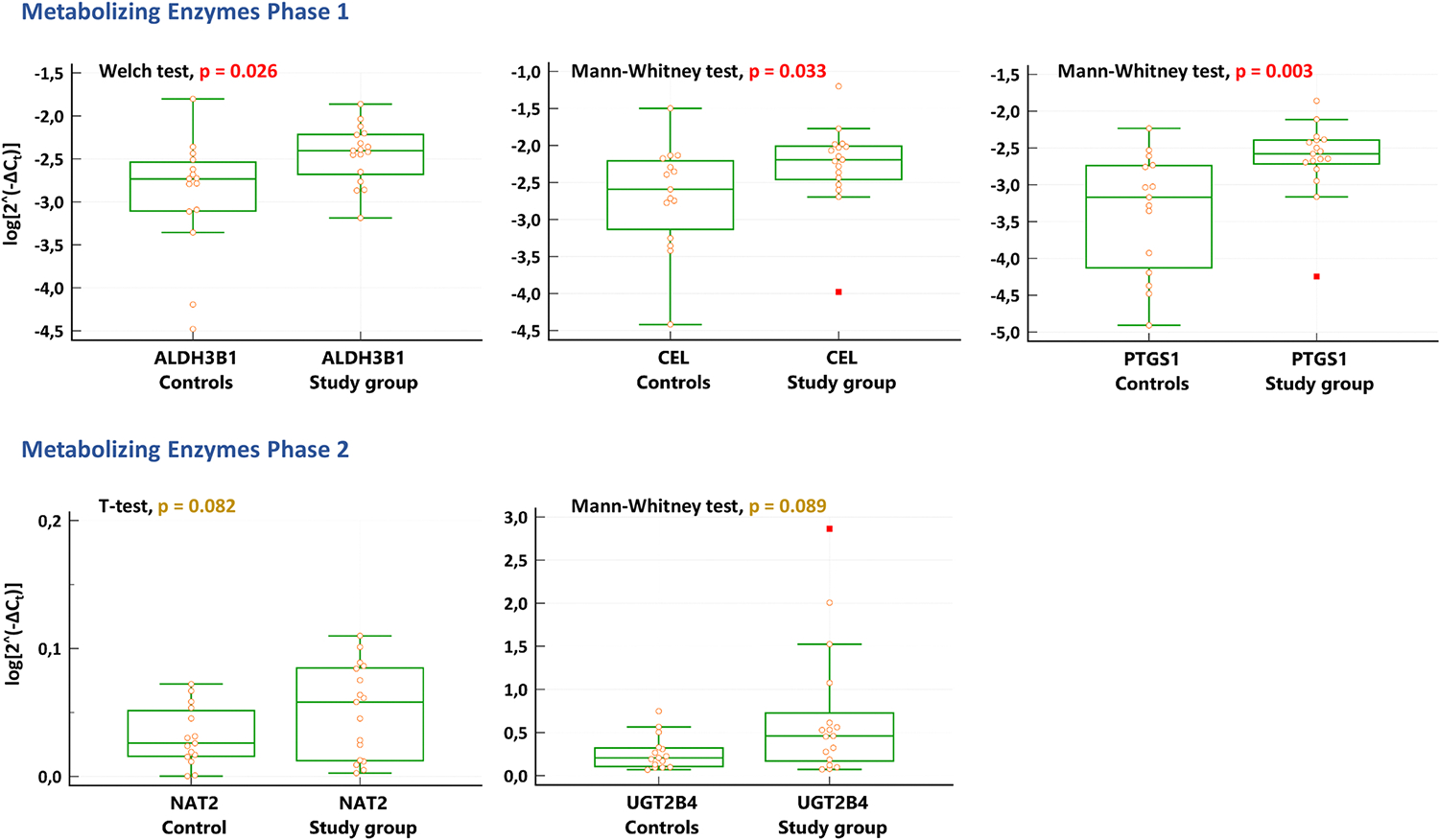
Differentially expressed genes of drug-metabolizing enzymes in human liver. Relative expression is shown as log-normalized [2^∧^(−ΔC_t_)] values. A box is drawn from the 1st to 3rd quartile, a horizontal line is drawn at the median. The upper inner fence is the 3rd quartile + 1.5 × IQR; The lower inner fence is the 1st quartile – 1.5 IQR. The upper whisker is drawn at the highest value (observation, measurement) just below the upper inner fence. The lower whisker is drawn at the highest value (observation, measurement) just above the lower inner fence. Statistical test used for comparison with the respective p-value is shown in each single graph.

Differentially expressed genes (DEGs) in the liver were *ALDH3B1*, *CEL,* and *PTGS1* of phase 1 enzymes, all up-regulated in the study group (Fig. 3); additionally, a trend towards higher hepatic mRNA expression was noted for *NAT2* and *UGT2B4* of phase 2 enzymes. Furthermore, Spearman rank correlation analysis of log-normalized [2^∧^(−ΔC_t_)] and BMI values was performed for all genes with potential biological relevance (Fig. 2). Significant correlation with BMI was noted for *ALDH3B1* (*rho* = 0.537; p = 0.002), *CEL* (*rho* = 0.372, p = 0.036), *CYP2D6* (*rho* = 0.362, p = 0.042), *PTGS1* (*rho* = 0.427, p = 0.015). Close to significant correlation with BMI was observed for *GALNT4* (*rho* = 0.304; p = 0.091), *GSTM2* (*rho* = 0.324, p = 0.071), *UGT2A3* (*rho* = 0.337, p = 0.059), *UGT2B4* (*rho* = 0.331, p = 0.064).

## 4. Discussion

Managing pharmacotherapy in individuals with obesity and its complications requires heightened attention due to the correlation between BMI and adverse health outcomes [1]. As BMI increases, the risk of various noncommunicable diseases increases, including cardiovascular diseases, diabetes, osteoarthritis, and some cancers [25]. This necessitates treatment, leading to increased medication intake and substantial healthcare costs. In the US alone, annual medical costs for individuals with obesity surpass those for lean individuals by over 1800 $ per person, totaling over $170 billion annually for obesity-related expenses and exceeding $200 billion when patients with overweight were included [26].

Obesity leads to physiological changes that may impact every ADME phase. The changes include gastrointestinal transit, gut permeability, disproportion in body water and fat, cardiac output, changes in organ sizes, and liver and kidney function; moreover, obesity is characterized by chronic low-grade inflammation [4,27]. These differences in PK can significantly affect the clinical efficacy and tolerance of drugs and, thus, require optimization of doses in the population with obesity [27]. For dose adjustment in patients with obesity, the following PK assumptions have been proposed: 1) the volume of distribution increases for lipophilic drugs but not for hydrophilic molecules; 2) lean body weight serves as a size descriptor and better predicts clearance and volume of distribution; 3) CYP3A4 activity is decreased, and UGTs is increased; 4) glomerular filtration rate is increased. Zhang et al. [28] challenged these common assumptions based on the existing evidence.

Our study focused on the underlying mechanisms of the above-mentioned differences in PK, which are still insufficiently described. We screened for differences in the mRNA expression of 168 genes encoding metabolizing enzymes (phases 1 and 2). Yet, our observations require further confirmation on the protein level.

### 4.1. Phase 1 metabolism

The CYP450 enzyme system has often garnered more attention since the three families CYP1, CYP2, and CYP3 account for up to 90 % of the total metabolism of xenobiotics [29]. Our work did not reveal any significant differences in mRNA expression of either studied CYP isoform (for the complete list of genes, see Table S1 and Fig. 1). However, the fold-change analysis (Figs. 1 and 2) showed potential biologically relevant down-regulation of *CYP2C19* (FC = −1.64) and up-regulation of *CYP2B6* (FC = 1.54), *CYP2D6* (FC = 1.61), and *CYP7A1* (FC = 8.34). Moreover, we observed a significant positive correlation between *CYP2D6* expression and BMI, which could support the literature data on increased CYP2D6 activity in obesity [27]. A study by Krogstad et al. analyzed the activity of major CYP450 isoforms in the liver and small intestine samples from patients with obesity: CYP1A2, CYP2B6, CYP2C8, CYP2C9, CYP2C19, CYP2D6, and CYP3A. They showed a negative correlation between hepatic CYP2B6 activity and BMI (the study cohort included 20 patients) [30]. The group did not confirm that on a larger cohort (n = 36) but found a significant correlation between CYP3A activity in the liver and the parameters defining body weight and composition (e.g., BMI, body weight, fat percent) [31]. Literature reviews do not entirely agree on the impact of obesity on CYP isoforms activity, indicating reduced activity of CYP3A4/5 [27,29] (inconclusive in [28]), CYP1A2 [29] (not confirmed by [27]), and CYP2C9 [29] (not confirmed by [27]), CYP2C19 [27] (inconclusive in [29]). At the same time, it increases the activity of CYP2D6 [27] (inconclusive in [29]) and CYP2E1 [29]. Our results on *CYP2C19* would be in line with findings reported by Kvitne et al. [32], which showed decreased CYP2C19-mediated metabolism in obesity. The CYP2C19-mediated metabolism increases after weight loss. The group did not find such differences for CYP2C9 or CYP1A2 [32]. Notably, studies by Kvitne et al. [18] evidenced that the protein expression cannot be directly translated into plasma clearance. They observed the lack of association between CYP3A4 protein expression in the liver and midazolam plasma clearance, what supports that hepatic blood flow might have a crucial role in determining midazolam clearance [18]. Similarly, van Rongen et al. concluded on increased CYP2E1-mediated clearance in obesity, while no difference was observed in CYP2E1 activity [33].

Nevertheless, phase 1 metabolism of xenobiotics is not limited to CYP450 and involves other enzymes such as aldehyde oxidases, aldehyde dehydrogenases (ALDHs), alcohol dehydrogenases, flavin-containing monooxygenases, monoamine oxidases, xanthine oxidoreductases, and others [34,35]. We found significantly higher (by 1.7-fold) expression of *ALDH3B1* mRNA in the liver of individuals with obesity/overweight, and a significant correlation between *ALDH3B1* expression and BMI. ALDH3B1 enzyme is one of 19 isoforms of human ALDHs and is localized in the cytosol but associated with the plasma membrane [34,36] of the liver, kidney, lung, and brain [37]. ALDHs oxidize toxic aldehydes to carboxyl metabolites, and several isoforms hydrolyze esters [38]. ALDH3B1 protects cells against aldehydes (from alcohol metabolism and lipid peroxidation) and oxidant cytotoxicity [36]. Therefore, the oxidative stress related to obesity [39] may be a reason for the observed up-regulation of *ALDH3B1* in the liver. To our knowledge, there is no information on the direct involvement of ALDH3B1 in drug metabolism. However, polymorphisms within the *ALDH3B1* gene were significantly associated with differences in simvastatin C_max_, and a particular genotype was hypothesized to relate to the decreased absorption of simvastatin [40].

Two isoforms of prostaglandin endoperoxide synthase, more commonly known as cyclooxygenase 1 (COX-1) and COX-2, are encoded by *PTGS1* and *PTGS2* genes, respectively. Both isoforms catalyze the same process of oxidative conversion of arachidonic acid to prostaglandin H_2_, but *PTGS1* is constitutively expressed, while *PTGS2* expression is markedly induced by inflammation. [41] Therefore, COX-1 provides an immediate response to inflammatory stimuli, whereas COX-2 contributes to prostanoid production as inflammation progresses [42]. Our study revealed the potentially relevant up-regulation of both genes in the liver (2.8-fold for *PTGS1* and 3.5-fold for *PTGS2*), but significance was reached only for *PTGS1* (Figs. 2 and 3). Additionally, a significant correlation was found between *PTGS1* expression and BMI. The observed overexpression can be associated with chronic low-grade inflammation, characteristic of obesity [4,27]. COX-2 was described to be an important contributor to obesity-related inflammation and to the development of non-alcoholic fatty liver disease – a disease that is strongly associated with obesity [43].

CEL (carboxyl ester lipase), also known as cholesterol esterase, is a lipolytic enzyme secreted by the pancreas and lactating mammary glands. Still, smaller amounts are also found, e.g., in the liver and endothelial cells. The physiological role of the enzyme is the hydrolysis of cholesterol and lipid-soluble vitamin esters to promote their digestion [44,45]. Our study observed higher (by 2-fold) hepatic mRNA expression of *CEL* (Figs. 2 and 3) and a significant correlation between the gene expression and BMI.

### 4.2. Phase 2 metabolism

Phase 2 of biotransformation includes processes of drug conjugation to hydrophilic endogenous substrates, e.g., glucuronidation, sulfation, and glutathione conjugation [46]. The major process is glucuronidation, which is catalyzed by UGTs. This group of 22 enzymes in humans is subdivided into four families: UGT1, UGT2, UGT3, and UGT8, but the contribution of UGT3 and UGT8 in drug metabolism is minimal [35]. Literature reviews pointed to an increased function of UGTs in individuals with obesity [27,28]. Our analysis comprised twelve enzymes from the UGT superfamily, including three members of UGT1 and seven UGT2 enzymes (Fig. 1); UGT1 and UGT2 families are primarily involved in drug metabolism [35]. Interestingly, potential biologically relevant up-regulation in the study group was found for most of the analyzed UGTs (Figs. 1 and 2), which aligns with the aforementioned information on increased glucuronidation in the population with obesity. However, the statistical significance was reached for neither of the genes. Only for the *UGT2B4* gene, a trend was observed towards overexpression (mRNA level) in the liver by 2.2-fold (Fig. 3). A correlation close to significant was observed between *UGT2B4* and *UGT2A3* expression and BMI. UGT2B4 enzyme is involved in the hepatic clearance of, e.g., clopidogrel carboxylic acid [47], carvedilol [48], and lorazepam [49]. The increased function of UGT2B4 could contribute to the lower AUC_0-inf_ of lorazepam observed in the population with obesity [49].

### 4.3. Study limitations

Our studies on the hepatic expression of a broad spectrum of genes encoding drug-metabolizing enzymes revealed several potential physiologically relevant differences. However, statistical significance was reached only for a few of them. This may result from the limited size of the studied cohort and the variability in relative expression data. A limitation of this study is that the expression analysis has been performed on post-mortem tissue, and therefore, possible natural RNA degradation has to be considered. However, factors have been discussed to influence RNA integrity and RT-PCR results, but normalization can minimize this effect [50]. Therefore, we carefully analyzed patient characteristics for any confounders (Table 1) and used two reference genes to avoid the possible influence of RNA integrity on the expression results. Also, the study population can be interpreted as a limitation but also as a strength. On one hand, the concomitant diseases may add to the variability of the expression results, but on the other, the control and study groups were well matched in terms of age, sex, and clinical conditions, and what should be emphasized is that they accurately reflect the real-life population.

### 4.4. Conclusions

Our study showed that only 3 out of 168 genes that have been analyzed were differentially expressed on mRNA level. Nevertheless, the alterations in expression observed at the mRNA level must be further explored at the protein level (abundance and function). Our study initially explored the expression changes and will facilitate the generation of additional hypotheses and allow us and others to continue confirmatory work. The PK pathways are complicated and they interplay; therefore, tremendous effort from researchers and many resources will be required to understand obesity-related alterations in PK, resulting in necessary clinical protocol adjustments.

## Supplementary Material

1

Appendix A. Supporting information

Supplementary data associated with this article can be found in the online version at doi:10.1016/j.biopha.2025.118155.

## Figures and Tables

**Table 1:** Clinical characteristics of the studied population.

Variables	Control group n = 15	Study group n = 17	p-value

Age (yrs)	66.0 (62.5 – 71.8)	65.0 (48.8 – 68.2)	NS
Sex			
Female (%)	40.0	41.2	NS
Male (%)	60.0	58.8	NS
BMI (kg/m^2^)	22.3 (21.1 – 24.0)	31.2 (27.7 – 37.3)	< 0.001
Body weight (kg)	64.6 ± 7.8	96.3 ± 26.9	< 0.001
Post-mortem interval (h)	13.6 ± 3.0	13.3 ± 3.2	NS
Race			
African American (%)	6.7	11.8	NS
Caucasian (%)	93.3	82.3	NS
Unknown (%)	0.0	5.9	NS
Concomitant diseases			
Asthma (%)	36.4	23.5	NS
COPD (%)	33.3	11.8	NS
Diabetes type 1 (%)	0	5.9	NS
Diabetes type 2 (%)	20.0	11.8	NS
Hypertension (%)	53.3	100.0	0.002
Heart failure (%)	13.3	11.8	NS
Thyroid disorder (%)	13.3	5.9	NS
Depression (%)	0	23.5	NS
Anxiety (%)	0	11.8	NS
Joint pain (%)	26.7	17.6	NS
CAD (%)	6.7	17.6	NS

CAD - Coronary Artery Disease; COPD - Chronic Obstructive Pulmonary Disease; NS – non-significant. Continuous data are expressed as mean ± SD or median (interquartile range). Normal distribution of data was tested with the Shapiro-Wilk test. Continuous data without normal distribution was compared using the Mann-Whitney *U* test. Data with normal distribution were compared with a T-test or a Welch test, depending on the equality of variances (F-test). Categorical data are expressed as percentages and were compared using a two-tailed Fisher’s exact test.

## Data Availability

Data will be made available on request.
