# Supplementary material for: Obesity-related drug-metabolizing enzyme expression alterations in the human liver
Source: Biomed Pharmacother. Author manuscript; Available in PMC 2025 Jun 2. (PMC12129139; doi:10.1016/j.biopha.2025.118155)
Supplement: 1 [file NIHMS2083343-supplement-1.docx]

**Table S1. List of all genes analyzed within the project** (Qiagen, cat. no. PAHS-068ZA and PAHS-069ZA)

| **No.** | **GenBank** | **Symbol** | **Description** |
| --- | --- | --- | --- |
| **Metabolizing Enzymes Phase 1 genes** | | | |
| 1 | NM_001086 | *AADAC* | Arylacetamide deacetylase (esterase) |
| 2 | NM_000667 | *ADH1A* | Alcohol dehydrogenase 1A (class I), alpha polypeptide |
| 3 | NM_000668 | *ADH1B* | Alcohol dehydrogenase 1B (class I), beta polypeptide |
| 4 | NM_000669 | *ADH1C* | Alcohol dehydrogenase 1C (class I), gamma polypeptide |
| 5 | NM_000670 | *ADH4* | Alcohol dehydrogenase 4 (class II), pi polypeptide |
| 6 | NM_000671 | *ADH5* | Alcohol dehydrogenase 5 (class III), chi polypeptide |
| 7 | NM_000672 | *ADH6* | Alcohol dehydrogenase 6 (class V) |
| 8 | NM_000673 | *ADH7* | Alcohol dehydrogenase 7 (class IV), mu or sigma polypeptide |
| 9 | NM_000689 | *ALDH1A1* | Aldehyde dehydrogenase 1 family, member A1 |
| 10 | NM_003888 | *ALDH1A2* | Aldehyde dehydrogenase 1 family, member A2 |
| 11 | NM_000693 | *ALDH1A3* | Aldehyde dehydrogenase 1 family, member A3 |
| 12 | NM_000692 | *ALDH1B1* | Aldehyde dehydrogenase 1 family, member B1 |
| 13 | NM_000690 | *ALDH2* | Aldehyde dehydrogenase 2 family (mitochondrial) |
| 14 | NM_000691 | *ALDH3A1* | Aldehyde dehydrogenase 3 family, member A1 |
| 15 | NM_000382 | *ALDH3A2* | Aldehyde dehydrogenase 3 family, member A2 |
| 16 | NM_000694 | *ALDH3B1* | Aldehyde dehydrogenase 3 family, member B1 |
| 17 | NM_000695 | *ALDH3B2* | Aldehyde dehydrogenase 3 family, member B2 |
| 18 | NM_003748 | *ALDH4A1* | Aldehyde dehydrogenase 4 family, member A1 |
| 19 | NM_001080 | *ALDH5A1* | Aldehyde dehydrogenase 5 family, member A1 |
| 20 | NM_005589 | *ALDH6A1* | Aldehyde dehydrogenase 6 family, member A1 |
| 21 | NM_001182 | *ALDH7A1* | Aldehyde dehydrogenase 7 family, member A1 |
| 22 | NM_022568 | *ALDH8A1* | Aldehyde dehydrogenase 8 family, member A1 |
| 23 | NM_000696 | *ALDH9A1* | Aldehyde dehydrogenase 9 family, member A1 |
| 24 | NM_001807 | *CEL* | Carboxyl ester lipase (bile salt-stimulated lipase) |
| 25 | NM_000781 | *CYP11A1* | Cytochrome P450, family 11, subfamily A, polypeptide 1 |
| 26 | NM_000497 | *CYP11B1* | Cytochrome P450, family 11, subfamily B, polypeptide 1 |
| 27 | NM_000498 | *CYP11B2* | Cytochrome P450, family 11, subfamily B, polypeptide 2 |
| 28 | NM_000102 | *CYP17A1* | Cytochrome P450, family 17, subfamily A, polypeptide 1 |
| 29 | NM_000103 | *CYP19A1* | Cytochrome P450, family 19, subfamily A, polypeptide 1 |
| 30 | NM_000499 | *CYP1A1* | Cytochrome P450, family 1, subfamily A, polypeptide 1 |
| 31 | NM_000761 | *CYP1A2* | Cytochrome P450, family 1, subfamily A, polypeptide 2 |
| 32 | NM_000104 | *CYP1B1* | Cytochrome P450, family 1, subfamily B, polypeptide 1 |
| 33 | NM_000500 | *CYP21A2* | Cytochrome P450, family 21, subfamily A, polypeptide 2 |
| 34 | NM_000782 | *CYP24A1* | Cytochrome P450, family 24, subfamily A, polypeptide 1 |
| 35 | NM_000783 | *CYP26A1* | Cytochrome P450, family 26, subfamily A, polypeptide 1 |
| 36 | NM_019885 | *CYP26B1* | Cytochrome P450, family 26, subfamily B, polypeptide 1 |
| 37 | NM_183374 | *CYP26C1* | Cytochrome P450, family 26, subfamily C, polypeptide 1 |
| 38 | NM_000784 | *CYP27A1* | Cytochrome P450, family 27, subfamily A, polypeptide 1 |
| 39 | NM_000785 | *CYP27B1* | Cytochrome P450, family 27, subfamily B, polypeptide 1 |
| 40 | NM_000766 | *CYP2A13* | Cytochrome P450, family 2, subfamily A, polypeptide 13 |
| 41 | NM_000767 | *CYP2B6* | Cytochrome P450, family 2, subfamily B, polypeptide 6 |
| 42 | NM_000772 | *CYP2C18* | Cytochrome P450, family 2, subfamily C, polypeptide 18 |
| 43 | NM_000769 | *CYP2C19* | Cytochrome P450, family 2, subfamily C, polypeptide 19 |
| 44 | NM_000770 | *CYP2C8* | Cytochrome P450, family 2, subfamily C, polypeptide 8 |
| 45 | NM_000771 | *CYP2C9* | Cytochrome P450, family 2, subfamily C, polypeptide 9 |
| 46 | NM_000106 | *CYP2D6* | Cytochrome P450, family 2, subfamily D, polypeptide 6 |
| 47 | NM_000773 | *CYP2E1* | Cytochrome P450, family 2, subfamily E, polypeptide 1 |
| 48 | NM_000774 | *CYP2F1* | Cytochrome P450, family 2, subfamily F, polypeptide 1 |
| 49 | NM_024514 | *CYP2R1* | Cytochrome P450, family 2, subfamily R, polypeptide 1 |
| 50 | NM_030622 | *CYP2S1* | Cytochrome P450, family 2, subfamily S, polypeptide 1 |
| 51 | NM_017781 | *CYP2W1* | Cytochrome P450, family 2, subfamily W, polypeptide 1 |
| 52 | NM_017460 | *CYP3A4* | Cytochrome P450, family 3, subfamily A, polypeptide 4 |
| 53 | NM_022820 | *CYP3A43* | Cytochrome P450, family 3, subfamily A, polypeptide 43 |
| 54 | NM_000777 | *CYP3A5* | Cytochrome P450, family 3, subfamily A, polypeptide 5 |
| 55 | NM_000765 | *CYP3A7* | Cytochrome P450, family 3, subfamily A, polypeptide 7 |
| 56 | NM_000778 | *CYP4A11* | Cytochrome P450, family 4, subfamily A, polypeptide 11 |
| 57 | NM_001010969 | *CYP4A22* | Cytochrome P450, family 4, subfamily A, polypeptide 22 |
| 58 | NM_000779 | *CYP4B1* | Cytochrome P450, family 4, subfamily B, polypeptide 1 |
| 59 | NM_021187 | *CYP4F11* | Cytochrome P450, family 4, subfamily F, polypeptide 11 |
| 60 | NM_023944 | *CYP4F12* | Cytochrome P450, family 4, subfamily F, polypeptide 12 |
| 61 | NM_001082 | *CYP4F2* | Cytochrome P450, family 4, subfamily F, polypeptide 2 |
| 62 | NM_000896 | *CYP4F3* | Cytochrome P450, family 4, subfamily F, polypeptide 3 |
| 63 | NM_007253 | *CYP4F8* | Cytochrome P450, family 4, subfamily F, polypeptide 8 |
| 64 | NM_000780 | *CYP7A1* | Cytochrome P450, family 7, subfamily A, polypeptide 1 |
| 65 | NM_004820 | *CYP7B1* | Cytochrome P450, family 7, subfamily B, polypeptide 1 |
| 66 | NM_004391 | *CYP8B1* | Cytochrome P450, family 8, subfamily B, polypeptide 1 |
| 67 | NM_182908 | *DHRS2* | Dehydrogenase/reductase (SDR family) member 2 |
| 68 | NM_000110 | *DPYD* | Dihydropyrimidine dehydrogenase |
| 69 | NM_001984 | *ESD* | Esterase D |
| 70 | NM_002021 | *FMO1* | Flavin containing monooxygenase 1 |
| 71 | NM_001460 | *FMO2* | Flavin containing monooxygenase 2 (non-functional) |
| 72 | NM_006894 | *FMO3* | Flavin containing monooxygenase 3 |
| 73 | NM_002022 | *FMO4* | Flavin containing monooxygenase 4 |
| 74 | NM_001461 | *FMO5* | Flavin containing monooxygenase 5 |
| 75 | NM_006144 | *GZMA* | Granzyme A (granzyme 1, cytotoxic T-lymphocyte-associated serine esterase 3) |
| 76 | NM_004131 | *GZMB* | Granzyme B (granzyme 2, cytotoxic T-lymphocyte-associated serine esterase 1) |
| 77 | NM_004493 | *HSD17B10* | Hydroxysteroid (17-beta) dehydrogenase 10 |
| 78 | NM_000240 | *MAOA* | Monoamine oxidase A |
| 79 | NM_000898 | *MAOB* | Monoamine oxidase B |
| 80 | NM_000962 | *PTGS1* | Prostaglandin-endoperoxide synthase 1 (prostaglandin G/H synthase and cyclooxygenase) |
| 81 | NM_000963 | *PTGS2* | Prostaglandin-endoperoxide synthase 2 (prostaglandin G/H synthase and cyclooxygenase) |
| 82 | NM_004181 | *UCHL1* | Ubiquitin carboxyl-terminal esterase L1 (ubiquitin thiolesterase) |
| 83 | NM_006002 | *UCHL3* | Ubiquitin carboxyl-terminal esterase L3 (ubiquitin thiolesterase) |
| 84 | NM_000379 | *XDH* | Xanthine dehydrogenase |
| **Metabolizing Enzymes Phase 2 genes** | | | |
| 85 | NM_001088 | *AANAT* | Aralkylamine N-acetyltransferase |
| 86 | NM_001995 | *ACSL1* | Acyl-CoA synthetase long-chain family member 1 |
| 87 | NM_004457 | *ACSL3* | Acyl-CoA synthetase long-chain family member 3 |
| 88 | NM_004458 | *ACSL4* | Acyl-CoA synthetase long-chain family member 4 |
| 89 | NM_052956 | *ACSM1* | Acyl-CoA synthetase medium-chain family member 1 |
| 90 | NM_182617 | *ACSM2B* | Acyl-CoA synthetase medium-chain family member 2B |
| 91 | NM_005622 | *ACSM3* | Acyl-CoA synthetase medium-chain family member 3 |
| 92 | NM_000030 | *AGXT* | Alanine-glyoxylate aminotransferase |
| 93 | NM_020682 | *AS3MT* | Arsenic (+3 oxidation state) methyltransferase |
| 94 | NM_004043 | *ASMT* | Acetylserotonin O-methyltransferase |
| 95 | NM_001701 | *BAAT* | Bile acid CoA: amino acid N-acyltransferase (glycine N-choloyltransferase) |
| 96 | NM_004059 | *CCBL1* | Cysteine conjugate-beta lyase, cytoplasmic |
| 97 | NM_001266 | *CES1* | Carboxylesterase 1 |
| 98 | NM_198061 | *CES2* | Carboxylesterase 2 |
| 99 | NM_024922 | *CES3* | Carboxylesterase 3 |
| 100 | NM_145024 | *CES5A* | Carboxylesterase 5A |
| 101 | NM_019886 | *CHST7* | Carbohydrate (N-acetylglucosamine 6-O) sulfotransferase 7 |
| 102 | NM_000754 | *COMT* | Catechol-O-methyltransferase |
| 103 | NM_005216 | *DDOST* | Dolichyl-diphosphooligosaccharide--protein glycosyltransferase |
| 104 | NM_000120 | *EPHX1* | Epoxide hydrolase 1, microsomal (xenobiotic) |
| 105 | NM_001979 | *EPHX2* | Epoxide hydrolase 2, cytoplasmic |
| 106 | NM_020474 | *GALNT1* | UDP-N-acetyl-alpha-D-galactosamine:polypeptide  N-acetylgalactosaminyltransferase 1 (GalNAc-T1) |
| 107 | NM_003774 | *GALNT4* | UDP-N-acetyl-alpha-D-galactosamine:polypeptide  N-acetylgalactosaminyltransferase 4 (GalNAc-T4) |
| 108 | NM_000156 | *GAMT* | Guanidinoacetate N-methyltransferase |
| 109 | NM_001490 | *GCNT1* | Glucosaminyl (N-acetyl) transferase 1, core 2 |
| 110 | NM_005838 | *GLYAT* | Glycine-N-acyltransferase |
| 111 | NM_018960 | *GNMT* | Glycine N-methyltransferase |
| 112 | NM_145740 | *GSTA1* | Glutathione S-transferase alpha 1 |
| 113 | NM_000847 | *GSTA3* | Glutathione S-transferase alpha 3 |
| 114 | NM_001512 | *GSTA4* | Glutathione S-transferase alpha 4 |
| 115 | NM_153699 | *GSTA5* | Glutathione S-transferase alpha 5 |
| 116 | NM_015917 | *GSTK1* | Glutathione S-transferase kappa 1 |
| 117 | NM_000848 | *GSTM2* | Glutathione S-transferase mu 2 (muscle) |
| 118 | NM_000849 | *GSTM3* | Glutathione S-transferase mu 3 (brain) |
| 119 | NM_000850 | *GSTM4* | Glutathione S-transferase mu 4 |
| 120 | NM_000851 | *GSTM5* | Glutathione S-transferase mu 5 |
| 121 | NM_004832 | *GSTO1* | Glutathione S-transferase omega 1 |
| 122 | NM_183239 | *GSTO2* | Glutathione S-transferase omega 2 |
| 123 | NM_000852 | *GSTP1* | Glutathione S-transferase pi 1 |
| 124 | NM_000853 | *GSTT1* | Glutathione S-transferase theta 1 |
| 125 | NM_006895 | *HNMT* | Histamine N-methyltransferase |
| 126 | NM_006774 | *INMT* | Indolethylamine N-methyltransferase |
| 127 | NM_002406 | *MGAT1* | Mannosyl (alpha-1,3-)-glycoprotein beta-1,2-N-acetylglucosaminyltransferase |
| 128 | NM_002408 | *MGAT2* | Mannosyl (alpha-1,6-)-glycoprotein beta-1,2-N-acetylglucosaminyltransferase |
| 129 | NM_020300 | *MGST1* | Microsomal glutathione S-transferase 1 |
| 130 | NM_002413 | *MGST2* | Microsomal glutathione S-transferase 2 |
| 131 | NM_004528 | *MGST3* | Microsomal glutathione S-transferase 3 |
| 132 | NM_016100 | *NAA20* | N(alpha)-acetyltransferase 20, NatB catalytic subunit |
| 133 | NM_000662 | *NAT1* | N-acetyltransferase 1 (arylamine N-acetyltransferase) |
| 134 | NM_000015 | *NAT2* | N-acetyltransferase 2 (arylamine N-acetyltransferase) |
| 135 | NM_006169 | *NNMT* | Nicotinamide N-methyltransferase |
| 136 | NM_000903 | *NQO1* | NAD(P)H dehydrogenase, quinone 1 |
| 137 | NM_000904 | *NQO2* | NAD(P)H dehydrogenase, quinone 2 |
| 138 | NM_002686 | *PNMT* | Phenylethanolamine N-methyltransferase |
| 139 | NM_017739 | *POMGNT1* | Protein O-linked mannose beta1,2-N-acetylglucosaminyltransferase |
| 140 | NM_004878 | *PTGES* | Prostaglandin E synthase |
| 141 | NM_002970 | *SAT1* | Spermidine/spermine N1-acetyltransferase 1 |
| 142 | NM_001055 | *SULT1A1* | Sulfotransferase family, cytosolic, 1A, phenol-preferring, member 1 |
| 143 | NM_177528 | *SULT1A2* | Sulfotransferase family, cytosolic, 1A, phenol-preferring, member 2 |
| 144 | NM_014465 | *SULT1B1* | Sulfotransferase family, cytosolic, 1B, member 1 |
| 145 | NM_001056 | *SULT1C2* | Sulfotransferase family, cytosolic, 1C, member 2 |
| 146 | NM_001008743 | *SULT1C3* | Sulfotransferase family, cytosolic, 1C, member 3 |
| 147 | NM_006588 | *SULT1C4* | Sulfotransferase family, cytosolic, 1C, member 4 |
| 148 | NM_005420 | *SULT1E1* | Sulfotransferase family 1E, estrogen-preferring, member 1 |
| 149 | NM_003167 | *SULT2A1* | Sulfotransferase family, cytosolic, 2A, dehydroepiandrosterone  (DHEA)-preferring, member 1 |
| 150 | NM_004605 | *SULT2B1* | Sulfotransferase family, cytosolic, 2B, member 1 |
| 151 | NM_014351 | *SULT4A1* | Sulfotransferase family 4A, member 1 |
| 152 | NM_001032377 | *SULT6B1* | Sulfotransferase family, cytosolic, 6B, member 1 |
| 153 | NM_000367 | *TPMT* | Thiopurine S-methyltransferase |
| 154 | NM_003312 | *TST* | Thiosulfate sulfurtransferase (rhodanese) |
| 155 | NM_003358 | *UGCG* | UDP-glucose ceramide glucosyltransferase |
| 156 | NM_000463 | *UGT1A1* | UDP glucuronosyltransferase 1 family, polypeptide A1 |
| 157 | NM_007120 | *UGT1A4* | UDP glucuronosyltransferase 1 family, polypeptide A4 |
| 158 | NM_021027 | *UGT1A9* | UDP glucuronosyltransferase 1 family, polypeptide A9 |
| 159 | NM_006798 | *UGT2A1* | UDP glucuronosyltransferase 2 family, polypeptide A1, complex locus |
| 160 | NM_024743 | *UGT2A3* | UDP glucuronosyltransferase 2 family, polypeptide A3 |
| 161 | NM_001075 | *UGT2B10* | UDP glucuronosyltransferase 2 family, polypeptide B10 |
| 162 | NM_001077 | *UGT2B17* | UDP glucuronosyltransferase 2 family, polypeptide B17 |
| 163 | NM_053039 | *UGT2B28* | UDP glucuronosyltransferase 2 family, polypeptide B28 |
| 164 | NM_021139 | *UGT2B4* | UDP glucuronosyltransferase 2 family, polypeptide B4 |
| 165 | NM_001074 | *UGT2B7* | UDP glucuronosyltransferase 2 family, polypeptide B7 |
| 166 | NM_152404 | *UGT3A1* | UDP glycosyltransferase 3 family, polypeptide A1 |
| 167 | NM_003360 | *UGT8* | UDP glycosyltransferase 8 |
| 168 | NM_000379 | *XDH* | Xanthine dehydrogenase |
| **Reference gene candidates** | | | |
| 169 | NM_001101 | *ACTB* | Actin, beta |
| 170 | NM_004048 | *B2M* | Beta-2-microglobulin |
| 171 | NM_002046 | *GAPDH* | Glyceraldehyde-3-phosphate dehydrogenase |
| 172 | NM_000194 | *HPRT1* | Hypoxanthine phosphoribosyltransferase 1 |
| 173 | NM_001002 | *RPLP0* | Ribosomal protein, large, P0 |
| 174 | NR_003286 | *RNA18S5* | RNA, 18S ribosomal 5 |
| 175 | NM_021130 | *PPIA* | peptidylprolyl isomerase A |

**Table S2. The mean [2^∧^(−ΔCt)] values and standard deviation (SD) for the analyzed genes**

| **No.** | **Gene symbol** | **control group (n = 15)** | | **study group (n = 17)** | |
| --- | --- | --- | --- | --- | --- |
|  |  | mean | SD | mean | SD |

| **Metabolizing Enzymes Phase 1 genes** |
| --- |

| 1 | *AADAC* | 4,49E-02 | 3,76E-02 | 5,70E-02 | 3,55E-02 |
| --- | --- | --- | --- | --- | --- |
| 2 | *ADH1A* | 7,64E-01 | 9,00E-01 | 8,37E-01 | 5,83E-01 |
| 3 | *ADH1B* | 3,94E+00 | 4,31E+00 | 3,34E+00 | 2,43E+00 |
| 4 | *ADH1C* | 1,92E+00 | 2,49E+00 | 1,42E+00 | 9,86E-01 |
| 5 | *ADH4* | 1,24E+00 | 1,26E+00 | 1,01E+00 | 1,03E+00 |
| 6 | *ADH5* | 2,67E-01 | 1,45E-01 | 2,73E-01 | 1,41E-01 |
| 7 | *ADH6* | 2,31E-01 | 2,71E-01 | 2,02E-01 | 1,84E-01 |
| 8 | *ALDH1A1* | 6,38E-01 | 4,47E-01 | 6,57E-01 | 3,59E-01 |
| 9 | *ALDH1A2* | 1,71E-03 | 1,40E-03 | 2,82E-03 | 2,53E-03 |
| 10 | *ALDH1A3* | 5,28E-03 | 4,15E-03 | 9,12E-03 | 1,05E-02 |
| 11 | *ALDH1B1* | 1,65E-02 | 1,78E-02 | 1,56E-02 | 1,37E-02 |
| 12 | *ALDH2* | 1,07E+00 | 9,77E-01 | 7,82E-01 | 4,20E-01 |
| 13 | *ALDH3A2* | 1,32E-01 | 8,12E-02 | 1,56E-01 | 9,14E-02 |
| 14 | *ALDH3B1* | 2,70E-03 | 3,70E-03 | 4,60E-03 | 3,19E-03 |
| 15 | *ALDH4A1* | 4,31E-01 | 1,90E-01 | 4,16E-01 | 2,70E-01 |
| 16 | *ALDH5A1* | 1,55E-01 | 1,73E-01 | 1,38E-01 | 9,18E-02 |
| 17 | *ALDH6A1* | 5,72E-01 | 3,60E-01 | 5,01E-01 | 2,65E-01 |
| 18 | *ALDH7A1* | 4,14E-01 | 3,37E-01 | 3,41E-01 | 1,95E-01 |
| 19 | *ALDH8A1* | 5,10E-02 | 2,98E-02 | 5,17E-02 | 2,87E-02 |
| 20 | *ALDH9A1* | 1,07E-01 | 5,94E-02 | 9,58E-02 | 3,34E-02 |
| 21 | *CEL* | 5,07E-03 | 7,56E-03 | 9,94E-03 | 1,39E-02 |
| 22 | *CYP11A1* | 3,32E-03 | 2,16E-03 | 2,89E-03 | 3,73E-03 |
| 23 | *CYP1A1* | 8,18E-02 | 1,50E-01 | 7,78E-02 | 1,04E-01 |
| 24 | *CYP1A2* | 3,23E-01 | 4,15E-01 | 3,15E-01 | 3,39E-01 |
| 25 | *CYP1B1* | 7,52E-03 | 1,19E-02 | 9,76E-03 | 1,27E-02 |
| 26 | *CYP21A2* | 5,53E-02 | 6,83E-02 | 6,23E-02 | 4,77E-02 |
| 27 | *CYP26A1* | 5,63E-03 | 9,74E-03 | 6,19E-03 | 9,64E-03 |
| 28 | *CYP27A1* | 5,00E-01 | 5,00E-01 | 5,20E-01 | 4,07E-01 |
| 29 | *CYP2A13* | 8,49E-02 | 8,91E-02 | 8,91E-02 | 9,96E-02 |
| 30 | *CYP2B6* | 3,01E-01 | 3,59E-01 | 4,64E-01 | 5,94E-01 |
| 31 | *CYP2C18* | 3,43E-02 | 1,29E-02 | 4,23E-02 | 2,70E-02 |
| 32 | *CYP2C19* | 5,20E-01 | 9,96E-01 | 3,18E-01 | 3,14E-01 |
| 33 | *CYP2C8* | 3,81E-01 | 2,47E-01 | 4,24E-01 | 3,13E-01 |
| 34 | *CYP2C9* | 4,76E-01 | 3,40E-01 | 7,02E-01 | 6,03E-01 |
| 35 | *CYP2D6* | 4,36E-01 | 4,62E-01 | 7,00E-01 | 5,90E-01 |
| 36 | *CYP2E1* | 1,06E+01 | 7,23E+00 | 1,05E+01 | 8,43E+00 |
| 37 | *CYP2R1* | 2,07E-02 | 1,36E-02 | 2,42E-02 | 1,70E-02 |
| 38 | *CYP3A4* | 1,15E+00 | 1,00E+00 | 1,28E+00 | 1,29E+00 |
| 39 | *CYP3A43* | 2,18E-02 | 4,63E-02 | 1,71E-02 | 3,46E-02 |
| 40 | *CYP3A5* | 2,32E+00 | 3,01E+00 | 1,88E+00 | 1,89E+00 |
| 41 | *CYP3A7* | 2,89E-02 | 4,66E-02 | 2,12E-02 | 2,71E-02 |
| 42 | *CYP4A11* | 4,84E-01 | 3,96E-01 | 5,74E-01 | 3,97E-01 |
| 43 | *CYP4A22* | 1,19E-01 | 1,37E-01 | 1,17E-01 | 1,32E-01 |
| 44 | *CYP4F11* | 2,63E-02 | 1,39E-02 | 3,18E-02 | 1,72E-02 |
| 45 | *CYP4F12* | 7,05E-02 | 8,11E-02 | 7,35E-02 | 5,71E-02 |
| 46 | *CYP4F2* | 2,54E-01 | 2,30E-01 | 3,17E-01 | 3,36E-01 |
| 47 | *CYP4F3* | 3,59E-01 | 3,34E-01 | 4,72E-01 | 3,09E-01 |
| 48 | *CYP7A1* | 6,62E-03 | 1,03E-02 | 5,52E-02 | 1,29E-01 |
| 49 | *CYP7B1* | 1,41E-02 | 9,73E-03 | 1,53E-02 | 7,16E-03 |
| 50 | *CYP8B1* | 2,28E-01 | 1,15E-01 | 2,84E-01 | 2,22E-01 |
| 51 | *DHRS2* | 2,85E-02 | 4,58E-02 | 1,28E-02 | 1,59E-02 |
| 52 | *DPYD* | 1,60E-01 | 1,28E-01 | 2,29E-01 | 1,94E-01 |
| 53 | *ESD* | 1,25E-01 | 8,08E-02 | 1,28E-01 | 4,25E-02 |
| 54 | *FMO2* | 5,40E-03 | 3,58E-03 | 1,05E-02 | 1,26E-02 |
| 55 | *FMO3* | 2,99E-01 | 1,70E-01 | 2,86E-01 | 2,09E-01 |
| 56 | *FMO4* | 3,46E-02 | 2,69E-02 | 4,10E-02 | 2,99E-02 |
| 57 | *FMO5* | 1,24E-01 | 8,89E-02 | 1,30E-01 | 8,08E-02 |
| 58 | *GZMA* | 8,39E-03 | 1,15E-02 | 6,90E-03 | 5,85E-03 |
| 59 | *GZMB* | 1,07E-02 | 1,43E-02 | 5,79E-03 | 6,66E-03 |
| 60 | *HSD17B10* | 7,13E-02 | 4,91E-02 | 5,39E-02 | 2,38E-02 |
| 61 | *MAOA* | 2,59E-01 | 1,67E-01 | 1,98E-01 | 8,10E-02 |
| 62 | *MAOB* | 2,53E-01 | 1,41E-01 | 2,80E-01 | 1,43E-01 |
| 63 | *PTGS1* | 1,24E-03 | 1,52E-03 | 3,45E-03 | 3,10E-03 |
| 64 | *PTGS2* | 2,57E-02 | 3,11E-02 | 9,01E-02 | 2,70E-01 |
| 65 | *UCHL1* | 2,60E-03 | 5,21E-03 | 1,46E-03 | 1,55E-03 |
| 66 | *UCHL3* | 4,03E-02 | 3,10E-02 | 4,25E-02 | 2,17E-02 |
| 67 | *XDH* | 5,29E-02 | 3,90E-02 | 6,34E-02 | 4,81E-02 |

| **Metabolizing Enzymes Phase 2 genes** |
| --- |

| 68 | *ACSL1* | 3,24E+00 | 1,49E+00 | 4,44E+00 | 2,38E+00 |
| --- | --- | --- | --- | --- | --- |
| 69 | *ACSL3* | 1,77E-01 | 7,48E-02 | 2,11E-01 | 9,24E-02 |
| 70 | *ACSL4* | 8,64E-02 | 1,14E-01 | 1,12E-01 | 1,31E-01 |
| 71 | *ACSM1* | 5,07E-03 | 5,97E-03 | 7,63E-03 | 9,59E-03 |
| 72 | *ACSM2B* | 2,25E-01 | 2,82E-01 | 4,34E-01 | 5,20E-01 |
| 73 | *ACSM3* | 1,17E-01 | 9,87E-02 | 1,38E-01 | 1,19E-01 |
| 74 | *AGXT* | 4,26E+00 | 2,13E+00 | 5,24E+00 | 3,50E+00 |
| 75 | *AS3MT* | 6,39E-02 | 4,49E-02 | 8,52E-02 | 6,40E-02 |
| 76 | *BAAT* | 4,59E+00 | 3,18E+00 | 6,43E+00 | 5,66E+00 |
| 77 | *KYAT1* | 4,16E-02 | 3,00E-02 | 4,90E-02 | 3,01E-02 |
| 78 | *CES1* | 6,24E+00 | 3,15E+00 | 8,26E+00 | 6,70E+00 |
| 79 | *CES2* | 1,94E+00 | 7,63E-01 | 1,92E+00 | 8,88E-01 |
| 80 | *CES3* | 1,10E-01 | 7,85E-02 | 1,89E-01 | 1,62E-01 |
| 81 | *CES5A* | 2,51E-02 | 2,63E-02 | 3,15E-02 | 2,98E-02 |
| 82 | *CHST7* | 1,44E-02 | 1,28E-02 | 4,24E-02 | 5,61E-02 |
| 83 | *COMT* | 9,89E-01 | 3,88E-01 | 8,80E-01 | 4,42E-01 |
| 84 | *DDOST* | 2,68E-01 | 1,16E-01 | 2,57E-01 | 7,70E-02 |
| 85 | *EPHX1* | 1,97E+00 | 1,02E+00 | 2,35E+00 | 1,96E+00 |
| 86 | *EPHX2* | 8,40E-01 | 4,71E-01 | 1,11E+00 | 9,18E-01 |
| 87 | *GALNT1* | 2,96E-01 | 1,78E-01 | 3,21E-01 | 1,62E-01 |
| 88 | *GALNT4* | 1,71E-02 | 1,36E-02 | 2,64E-02 | 2,17E-02 |
| 89 | *GAMT* | 1,62E+00 | 8,96E-01 | 1,19E+00 | 7,39E-01 |
| 90 | *GCNT1* | 2,22E-02 | 4,81E-02 | 2,22E-02 | 2,27E-02 |
| 91 | *GLYAT* | 4,40E-01 | 4,50E-01 | 5,33E-01 | 4,88E-01 |
| 92 | *GNMT* | 2,06E-01 | 2,60E-01 | 2,26E-01 | 2,75E-01 |
| 93 | *GSTA1* | 4,25E+00 | 3,92E+00 | 4,90E+00 | 5,69E+00 |
| 94 | *GSTA3* | 5,10E-02 | 5,28E-02 | 7,58E-02 | 1,01E-01 |
| 95 | *GSTA4* | 3,80E-02 | 2,40E-02 | 4,87E-02 | 4,12E-02 |
| 96 | *GSTK1* | 1,51E+00 | 4,97E-01 | 1,81E+00 | 1,37E+00 |
| 97 | *GSTM2* | 8,95E-02 | 6,44E-02 | 1,37E-01 | 1,17E-01 |
| 98 | *GSTM3* | 3,91E-02 | 3,28E-02 | 2,61E-02 | 2,47E-02 |
| 99 | *GSTM4* | 1,63E-01 | 9,83E-02 | 2,06E-01 | 2,24E-01 |
| 100 | *GSTM5* | 1,55E-02 | 2,26E-02 | 1,19E-02 | 1,15E-02 |
| 101 | *GSTO1* | 1,55E+00 | 6,25E-01 | 1,35E+00 | 4,81E-01 |
| 102 | *GSTO2* | 1,18E-02 | 1,20E-02 | 1,47E-02 | 1,29E-02 |
| 103 | *GSTP1* | 6,68E-02 | 5,27E-02 | 9,40E-02 | 6,96E-02 |
| 104 | *GSTT1* | 3,32E-01 | 2,58E-01 | 4,69E-01 | 3,50E-01 |
| 105 | *HNMT* | 4,23E-03 | 4,34E-03 | 6,48E-03 | 5,44E-03 |
| 106 | *INMT* | 7,27E-02 | 7,81E-02 | 1,07E-01 | 8,16E-02 |
| 107 | *MGAT1* | 1,45E-01 | 8,34E-02 | 1,73E-01 | 1,07E-01 |
| 108 | *MGAT2* | 2,82E-02 | 1,93E-02 | 2,60E-02 | 1,80E-02 |
| 109 | *MGST1* | 5,63E-01 | 4,08E-01 | 6,14E-01 | 3,23E-01 |
| 110 | *MGST2* | 5,22E-01 | 1,70E-01 | 6,06E-01 | 2,62E-01 |
| 111 | *MGST3* | 2,68E-01 | 1,11E-01 | 2,30E-01 | 8,91E-02 |
| 112 | *NAA20* | 9,51E-02 | 3,65E-02 | 1,00E-01 | 3,35E-02 |
| 113 | *NAT1* | 3,65E-03 | 4,98E-03 | 6,22E-03 | 6,81E-03 |
| 114 | *NAT2* | 5,05E-02 | 3,59E-02 | 7,83E-02 | 5,48E-02 |
| 115 | *NNMT* | 3,51E-02 | 3,70E-02 | 2,65E-02 | 1,89E-02 |
| 116 | *NQO1* | 1,91E-02 | 4,72E-02 | 1,42E-02 | 1,71E-02 |
| 117 | *NQO2* | 2,24E-01 | 7,80E-02 | 2,75E-01 | 1,71E-01 |
| 118 | *POMGNT1* | 2,06E-01 | 1,52E-01 | 2,64E-01 | 1,92E-01 |
| 119 | *SAT1* | 2,62E+00 | 1,16E+00 | 2,37E+00 | 1,06E+00 |
| 120 | *SULT1A1* | 4,62E-01 | 3,38E-01 | 4,00E-01 | 2,91E-01 |
| 121 | *SULT1A2* | 1,58E-01 | 1,12E-01 | 1,96E-01 | 2,52E-01 |
| 122 | *SULT1B1* | 1,44E-01 | 1,17E-01 | 1,67E-01 | 1,34E-01 |
| 123 | *SULT1C2* | 8,51E-03 | 1,81E-02 | 8,58E-03 | 1,38E-02 |
| 124 | *SULT1C4* | 2,40E-02 | 5,07E-02 | 1,46E-02 | 1,17E-02 |
| 125 | *SULT1E1* | 1,43E-01 | 3,27E-01 | 1,72E-01 | 2,73E-01 |
| 126 | *SULT2A1* | 3,11E+00 | 1,57E+00 | 4,19E+00 | 3,51E+00 |
| 127 | *TPMT* | 6,87E-02 | 4,77E-02 | 8,35E-02 | 5,87E-02 |
| 128 | *TST* | 9,63E-01 | 3,73E-01 | 9,36E-01 | 5,46E-01 |
| 129 | *UGCG* | 9,32E-02 | 3,53E-02 | 1,29E-01 | 7,24E-02 |
| 130 | *UGT1A1* | 3,12E-01 | 2,77E-01 | 4,96E-01 | 4,31E-01 |
| 131 | *UGT1A4* | 2,93E-01 | 2,49E-01 | 3,62E-01 | 2,63E-01 |
| 132 | *UGT1A9* | 1,82E-01 | 1,50E-01 | 1,97E-01 | 1,96E-01 |
| 133 | *UGT2A3* | 4,30E-02 | 3,62E-02 | 6,51E-02 | 4,91E-02 |
| 134 | *UGT2B10* | 2,77E-01 | 3,13E-01 | 5,53E-01 | 5,90E-01 |
| 135 | *UGT2B17* | 1,73E-01 | 1,40E-01 | 3,58E-01 | 3,63E-01 |
| 136 | *UGT2B28* | 1,49E+00 | 1,43E+00 | 2,61E+00 | 2,95E+00 |
| 137 | *UGT2B4* | 4,14E-01 | 2,42E-01 | 9,16E-01 | 7,63E-01 |
| 138 | *UGT2B7* | 1,78E+00 | 1,29E+00 | 2,78E+00 | 2,30E+00 |
| 139 | *UGT3A1* | 3,19E-02 | 2,40E-02 | 3,30E-02 | 2,61E-02 |
| 140 | *XDH* | 1,46E-01 | 9,39E-02 | 2,09E-01 | 1,71E-01 |
